# Comparative effectiveness of individualized longer and standardized shorter regimens in the treatment of multidrug resistant tuberculosis in a high burden country

**DOI:** 10.3389/fphar.2022.973713

**Published:** 2022-09-06

**Authors:** Abdul Wahid, Abdul Ghafoor, Abdul Wali Khan, Yaser Mohammed Al-Worafi, Abdullah Latif, Nisar Ahmed Shahwani, Muhammad Atif, Fahad Saleem, Nafees Ahmad

**Affiliations:** ^1^ Department of Pharmacy Practice, Faculty of Pharmacy and Health Sciences, University of Balochistan, Quetta, Pakistan; ^2^ National TB Control Program, Islamabad, Pakistan; ^3^ Department of Clinical Pharmacy, University of Science and Technology of Fujairah, Fujairah, United Arab Emirates; ^4^ Department of Pharmaceutical Chemistry, Faculty of Pharmacy and Health Sciences, University of Balochistan, Quetta, Pakistan; ^5^ Department of Pharmacy Practice, Faculty of Pharmacy, The Islamia University of Bahawalpur, Bahawalpur, Pakistan

**Keywords:** cure, death, longer treatment regimen, MDR-TB, shorter treatment regimen, sputum culture conversion

## Abstract

**Objective:** To compare the effectiveness of second line injectables containing shorter (duration 9–12 months) and longer treatment regimens (LTR, duration ≥ 20 months) among multidrug-resistant tuberculosis (MDR-TB) patients with no documented resistance and history of treatment with any second-line anti-TB drug (SLD) for ≥ 1 month.

**Methods:** This was an observational cohort study of MDR-TB patients treated at eight PMDT units in Pakistan. Patients’ data from baseline until treatment outcomes were collected from Electronic Nominal Recording and Reporting System. The treatment outcomes of “cured” and “treatment completed” were grouped together as successful, whereas “death,” “treatment failure,” and “lost to follow-up” were collectively grouped as unsuccessful outcomes. Time to sputum culture conversion (SCC) was analyzed using the Kaplan–Meier method and the differences between groups were compared through the log-rank test. Multivariate Cox proportional hazards and binary logistic regression analyses were used to find predictors of time to SCC and unsuccessful treatment outcomes. A *p*-value < 0.05 was considered statistically significant.

**Results:** A total 701 eligible MDR-TB patients [313 treated with shorter treatment regimen (STR) and 388 treated with LTR at eight centres in Pakistan were evaluated]. Time to achieve SCC was significantly shorter in STR group [mean: 2.03 months, 95% confidence interval (CI):1.79–2.26] than in LTR group (mean: 2.69 months, 95% CI: 2.35–3.03) (*p*-value<0.001, Log-rank test). Treatment success was higher in STR (83.7%) than in LTR (73.2%) group (*p*-value <0.001) due to high cure (79.9% vs. 70.9%, *p*-value = 0.006) and low death (9.9% vs. 18.3%, *p*-value = 0.002) rates with STR. Treatment with STR emerged the only predictor of early SCC [adjusted Hazards ratio (aHR) = 0.815, *p*-value = 0.014], whereas, patient’s age of 41–60 (OR = 2.62, *p*-value<0.001) and >60 years (OR = 5.84, *p*-value<0.001), baseline body weight of 31–60 (OR = 0.36, *p*-value = 0.001) and >60 kg (OR = 0.23, *p*-value <0.001), and treatment with LTR (OR = 1.88, *p*-value = 0.001) had statistically significant association with unsuccessful treatment outcomes.

**Conclusion:** STR exhibited superior anti-microbial activity against MDR-TB. When compared LTR, treatment with STR resulted in significantly early SCC, high cure, and lower death rates among MDR-TB patients who had no documented resistance and history of treatment with any SLD ≥ 1 month.

## Introduction

Although tuberculosis (TB) is a curable disease, but with an estimated 10 million cases and 1.5 million deaths in 2020, it is still the leading lethal bacterial infectious disease globally (Annabel et al., 2019). Along with reduced access to TB diagnosis and treatment, the incidence of multidrug-resistant TB defined as “TB caused by *Mycobacterium tuberculosis* (MTB) concurrently resistant to both rifampicin (R) and isoniazid (H), the two most effective first-line anti-TB drugs” remains a public health crisis, health security threat and a major obstacle to the effective control and eradication of TB (Ahmad et al., 2018; Annabel et al., 2019). It has been reported that an estimated 3%–4% of the new and 18%–21% of the previously treated TB patients suffer from MDR-TB (Annabel et al., 2019). As patients with MDR-TB require extensive chemotherapy for prolonged periods with less effective, more toxic, and expensive second-line anti-TB drugs (SLD) (WHO, 2011; WHO, 2014; Ahmad et al., 2018; Lan et al., 2020), they are at a substantial risk of unsuccessful treatment outcomes (41% in MDR-TB vs. 14% in drug-susceptible TB) (Annabel et al., 2019; WHO, 2020). Furthermore, the average treatment cost per MDR-TB patient is USD ≥ 1,000 vs. USD 40 per drug-susceptible TB patient (Annabel et al., 2019).

In order to improve the treatment outcomes of MDR-TB, reduce the incidence of adverse events and treatment cost, and improve the patients’ health related quality of life, different treatment regimens have been introduced over the last decade. In 2011, World Health Organization (WHO) recommended the conventional longer treatment regimen (LTR) for treating MDR-TB. For patients with no resistance to any SLD and no documented history of SLD use for ≥ 1 month, LTR comprised of at least 8 months treatment with *amikacin (Am)/kanamycin (Km)/capreomycin (Cm)* + *levofloxacin (Lfx)* + *ethionamide (Eto)* + *cycloserine (Cs)* + *pyrazinamide (Z)* and 12 months treatment with *Lfx* + *Eto* + *Cs* + *Z.* If a patient had resistance to any SLD or had documented history of SLD use for ≥ 1 month, it was suggested to add *para-amino salicylic acid (PAS)* to the abovementioned regimen (WHO, 2011; Naz et al., 2021). However, LTR had the drawbacks of poor treatment success rate, 56% globally and 45%–76.9% in various individual cohorts around the world (Ahuja et al., 2012; Carroll et al., 2012; Jain et al., 2014; Sagwa et al., 2014; Ahmad et al., 2015; Hoa et al., 2015; Aibana et al., 2017; Alene et al., 2017; El Hamdouni et al., 2019; Khan et al., 2019; Leveri et al., 2019; Atif et al., 2020; Lan et al., 2020; Phu et al., 2020), acquisition of additional drug resistance during treatment, prolonged treatment duration (≥20 months), being expensive and high incidence of clinically significant adverse events (Ahuja et al., 2012; Carroll et al., 2012; Jain et al., 2014; Sagwa et al., 2014; Ahmad et al., 2015; Hoa et al., 2015; Aibana et al., 2017; Alene et al., 2017; El Hamdouni et al., 2019; Khan et al., 2019; Leveri et al., 2019; Atif et al., 2020; Phu et al., 2020). In order to overcome these drawbacks, in 2016, WHO introduced 9–12 months standardized shorter treatment regimen (STR) also known as “Bangladesh regimen” (Van Deun et al., 2010; Aung et al., 2014; WHO, 2016). It comprised of treating MDR-TB patients for 4–6 months with *Km* + *moxifloxacin (Mfx)* + *prothionamide (Pto)* + *clofazimine (Cfz)* + *Z* + *ethambutol (E)* + *high dose H* followed by 5 months treatment with Mfx + *Cfz* + *Z* + *E* (Van Deun et al., 2010; Aung et al., 2014; WHO, 2016). The use of STR under operational conditions have produced comparatively better treatment success rates (80%–89%) (Van Deun et al., 2010; Aung et al., 2014; Piubello et al., 2014; Kuaban et al., 2015; Khan et al., 2017; Trébucq et al., 2018; Abidi et al., 2020; Wahid et al., 2021). However, for MDR-TB patients to be treated with STR, they should have no history of treatment with any SLD for ≥ 1 month, no resistance to any SLD in STR and no intolerance or risk of toxicity to any drug included in STR. Furthermore, patients with anyone of the following characteristics i.e., clinically severe disseminated or extra-pulmonary TB like tubercular meningitis, bilateral lung cavitation or lung lesions in more than three zones, co-infected with Human Immunodeficiency Virus (HIV), patients with alanine transaminase level > 5 times of upper normal limit, creatinine clearance < 50 ml/min, the corrected QT interval >500 ms or those that are pregnant are also ineligible for treatment with STR (WHO, 2016; Wahid et al., 2021). This means that patients treated with STR have comparatively less severe disease and suffer from less complicated MDR-TB than those treated with LTR. One of the studies which led to the WHO recommendation of using STR for MDR-TB treatment was the non-inferiority trial of Standard Treatment Regimen of Anti-Tuberculosis Drugs for Patients with MDR-TB (STREAM) (Nunn et al., 2014). However, the results of STREAM revealed that in patients with MDR-TB susceptible to aminoglycosides and fluoroquinolones, both STR and LTR performed well with 80% and 79% successful outcomes, respectively (Nunn et al., 2019). As lost to follow-up (LTFU) is one of the major causes of lower treatment success with LTR under operational conditions, the findings of STREAM trial advocated that better patient support and backing during treatment improves treatment outcomes, irrespective of regimen composition (Nunn et al., 2019). An IPD meta-analysis of MDR-TB patients treated with STR and LTR has concluded that standardised STR was associated with substantially less LTFU, and more failure/relapse in the presence of resistance to component medications. However, its findings should be interpreted with the potential of residual confounding bias, especially because most STR and LTR data included were from low or low-middle income and high or upper-middle income countries. Programs in the later settings would have greater resources and better management of adverse events and comorbidities such as HIV, which could contribute to better outcomes (Abidi et al., 2020).

In terms of MDR-TB burden, Pakistan currently ranks fifth globally and have 33 functional units for the programmatic management of drug-resistant TB (PMDT). In Pakistan, the treatment of MDR-TB with LTR was started back in 2010 and has resulted in a variable success rate of 40.5%–76.9% at different centres (Ahmad et al., 2015; Javaid et al., 2017a; Javaid et al., 2017b; Khan et al., 2019; Atif et al., 2020), whereas, the treatment of eligible MDR-TB patients with STR was initiated in 2017 and has produced a treatment success rate of 83.7% (Naz et al., 2021; Wahid et al., 2021). However, there is scarcity of published data on comparative effectiveness of STR and LTR among confounders matched MDR-TB patients treated under programmatic conditions with same resources from both Pakistan and elsewhere. Therefore, this study was conducted to evaluate the comparative effectiveness of LTR and STR among patients with MDR-TB at multiple PMDT units in Pakistan while we attempted to match for confounders.

## Materials and methods

### Study settings, subjects, and sample size calculation

**FIGURE 1:**
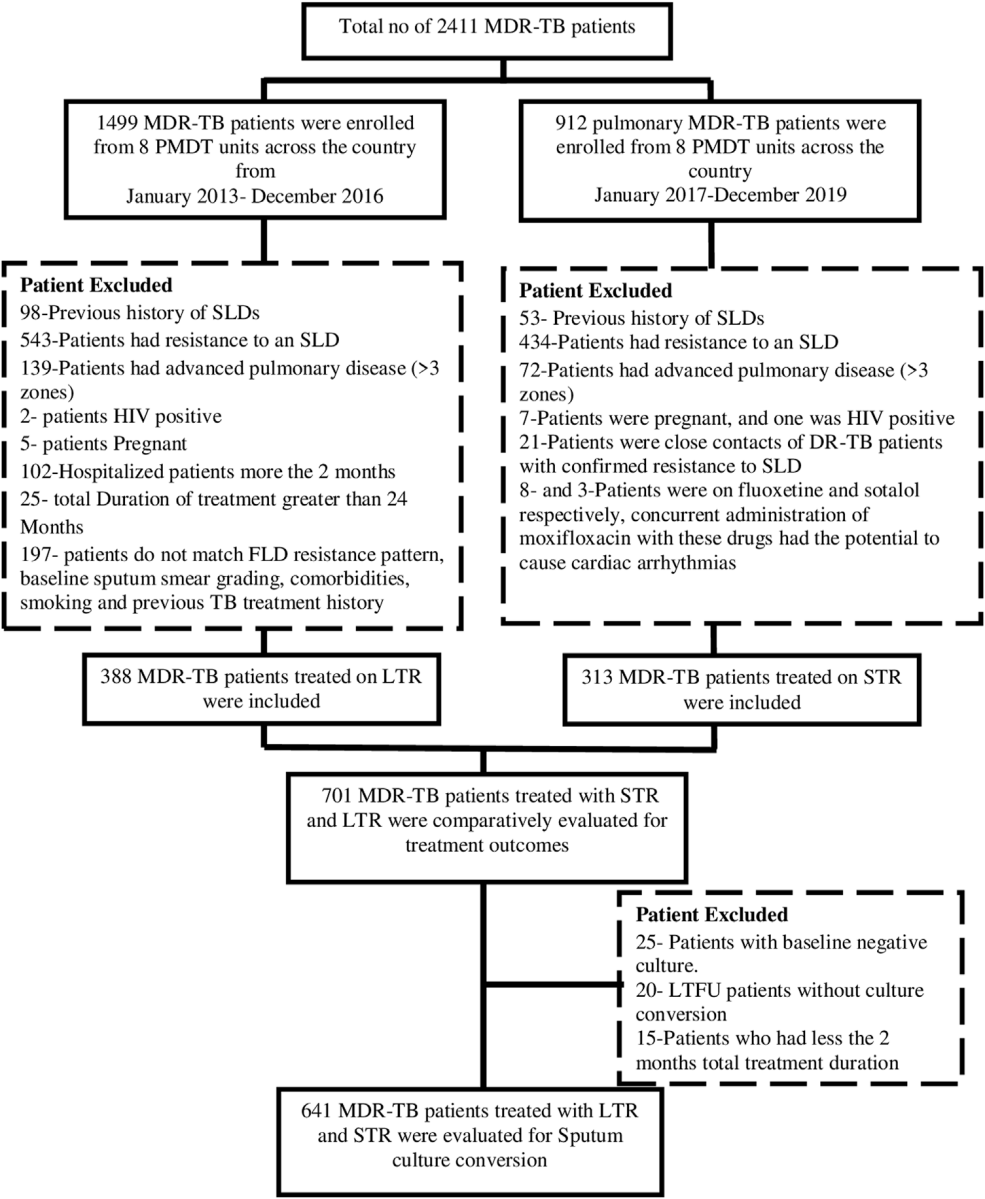
Fow chart of study participants included in the study SLDs, Second-line drugs; STR, Shorter treatment regimen; LTR, Longer treatment regimen; LTFU, Lost to follow-up; MDR-TB, Multi-drug resistant tuberclosis.

This was a retrospective cohort study carried out at the following PMDT units (Supplementary Table S1). All microbiologically confirmed 313 pulmonary MDR-TB patients who were treated with STR at these centers between December 2017 and July 2019 were included. The treatment outcomes of these STR treated patients as a separate cohort has already been published elsewhere (Wahid et al., 2021). For comparing the effectiveness of STR with LTR, a total of 3,88/1,499 microbiologically confirmed pulmonary MDR-TB patients who were enrolled at the study sites between 2013–2016 with no resistance to any SLD or history of treatment with any SLD ≥ 1 month, no bilateral lung cavitation or lung lesions in more than three zones, non-pregnant, not hospitalized for more than ≥ 1 month and HIV-negative patients were included in the study. The flowchart of enrollment of study participants is given in Figure 1.

### Diagnosis and treatment of study participants

The diagnosis and treatment of pulmonary MDR-TB patients with both LTR and STR at PMDT units in Pakistan have previously been reported elsewhere (Ahmad et al., 2015; Javaid et al., 2017c; Khan et al., 2019; Wahid et al., 2021). In compliance with the guidelines, two sputum samples of every presumed MDR-TB patient presented to a PMDT unit were initially evaluated for MTB infection and R and INH resistance by direct sputum smear microscopy using Ziehl–Neelsen staining, Xpert MTB/Rif (Cepheid, Sunnyvale, CA, United States) and line probe assay (2018 onward). After initial diagnosis of RR-TB, patients’ sputum samples were sent to reference laboratories where culture and phenotypic drug susceptibility (DST) against anti-TB drugs were carried out by Agar proportion method on enriched Middlebrook 7H10 medium (BBL; Beckton Dickinson, Sparks, MD, United States) at the concentrations given below: *R* [1 μg (µg)/ml (ml)], H (0.2 μg/ml), *streptomycin* (2 μg/ml), *ethambutol (E)* (5 μg/ml), *Am* (4 μg/ml), *Km* (5 μg/ml), *Cm* (4 μg/ml), Eto (5 μg/ml), *ofloxacin (Ofx)* (2 μg/ml) and *Lfx* (1 μg/ml). Whereas DST for Z was conducted at a concentration of 100 μg/ml using BACTEC Mycobacterial Growth Indicator Tube (MGIT, BD, Sparks, MD, United States). Before January 2017, all MDR-TB patients were treated with LTR, whereas, after January 2017, eligible MDR-TB patients were treated with STR, and the rest were treated with LTR. In the current study, LTR comprised of at least 8 months treatment with *Am/Km/Cm* + *Lfx* + *Eto* + *Cs* + *Z* and 12 months treatment with *Lfx* + *Eto* + *Cs* + *Z,* whereas the STR comprised of 4–6 months treatment with standard dose of *Km* + *Mfx* + *Pto* + *Cfz* + *Z* + *E* + *high dose H* followed by 5 months treatment with Mfx + *Cfz* + *Z* + *E* (Van Deun et al., 2010; Aung et al., 2014; WHO, 2016). Patients were given the maximum recommended doses of drugs per body weight.

### Data collection

Electronic nominal, recording and reporting System (ENRS) is a combined excel sheet containing information about the patients’ sociodemographic, microbiological, and clinical characteristics, treatment regimen and outcomes. All PMDT units use it to share MDR-TB patients’ data with national tuberculosis control program (NTP) every month. We retrieved study participants’ baseline and follow-up information from treatment initiation until the end of treatment from ENRS. Treatment outcomes of the current study participants were based on definitions given in WHO and NTP guidelines. The outcomes of “cured” and “treatment completed” were grouped as successful outcomes, whereas “death,” “LTFU” and “treatment failure” were grouped as unsuccessful outcomes.

### Data analysis

Statistical Package for Social Sciences (SPSS ver 26) was used for analysing data. Chi-square and Student’s *t*-test wherever applicable were used to compare the baseline characteristics of the patients treated with LTR and STR. In order to evaluate time to SCC defined as “the time from the data of initiating MDR-TB treatment to the collection date of the first two successive negative sputum cultures taken at least 1 month apart” (Basit et al., 2014; Javaid et al., 2018). All those patients who were sputum culture negative as the baseline visit, were LTFU prior to achieving SCC and had a total treatment duration of <2 months were excluded. Time to SCC was analysed by Kaplan Meier method, and differences between groups were assessed using the log-rank test. Multivariate Cox Proportional Hazards and binary logistic regression analyses were respectively used to evaluate predictors of time to SCC and unsuccessful treatment outcomes (Abubakar et al., 2022). After checking for correlation, those factors which had a *p*-value of < 0.2 in univariate analysis were entered in multivariate analysis. Findings with a *p*-value < 0.05 were considered statistically significant.

## Results

The baseline sociodemographic and clinical characteristics of the 701 patients (388 treated with LTR and 313 treated with STR) included in the final analysis are given in Table 1. There was no statistically significant difference in the patients of both groups in terms of gender, age, smoking, weight, sputum smear grading, comorbidity, lungs lesions, previous anti-TB treatment category and resistance pattern against FLD on baseline visit (*p*-value > 0.05) (Table 1).

**TABLE 1:** Patients’ baseline socio-demographic and clinical characteristics N = 701.

Variables	STR (%)	LTR (%)	*p*-value
Gender			
Female	157 (50.2)	199 (51.3)	0.766
Male	156 (49.8)	189 (48.7)	
Age (years)			
Mean ± SD 3.62 ± 15.65			
≤20	77 (24.6)	90 (23.2)	0.636
21–40	142 (45.4)	177 (55.5)	
41–60	67 (21.4)	95 (24.5)	
>60	27 (8.6)	26 (6.7)	
Smoking			
No	282 (90.1)	338 (87.1)	0.219
Yes	31 (9.9)	50 (12.9)	
Weight at base line			
≤30	16 (5.1)	28 (7.2)	0.198
31–60	256 (81.8)	323 (83.2)	
>60	41 (13.1)	37 (9.5)	
Sputum Smear			
Negative	31 (9.9)	37 (9.5)	0.984
Scanty + 1	176 (44.7)	218 (55.3)	
+2, +3	106 (33.9)	133 (34.3)	
Comorbidity			
No	256 (81.8)	307 (79.1)	0.378
Yes	57 (18.2)	81 (20.9_)	
Lung’s cavity			
No lesion	15 (4.8)	26 (6.7)	0.082
One zone	113 (36.1)	158 (40.7)	
2–3 zone	175 (55.9)	200 (51.5)	
Not available	10 (3.32)	4 (1.0)	
Previous TB treatment			
New	94 (30.0)	116 (29.9)	0.651
Cat-I	178 (56.9)	215 (55.4)	
Cat-II	19 (6.1)	33 (8.5)	
Unknown	22 (7.0)	24 (6.2)	
Resistance E			
No	270 (86.3)	317 (81.7)	0.122
Yes	43 (13.7)	71 (18.3)	
Resistance Z			
No	243 (77.6)	295 (76.0)	0.617
Yes	70 (22.4)	93 (24.0)	
Resistance S			
No	287 (91.7)	339 (87.4)	0.066
Yes	26 (8.3)	49 (12.6)	
Resist to all 5 FLD			
No	306 (97.8)	371 (95.6)	0.121
Yes	7 (2.2)	17 (4.4)	

TB, tuberculosis; STR, shorter treatment regimen; LTR, longer treatment regimen; FLD, first-line drugs; Z, pyrazinamide; E, ethambutol; S, streptomycin.

### Time to sputum culture conversion and predictors of early sputum culture conversion

**FIGURE 2:**
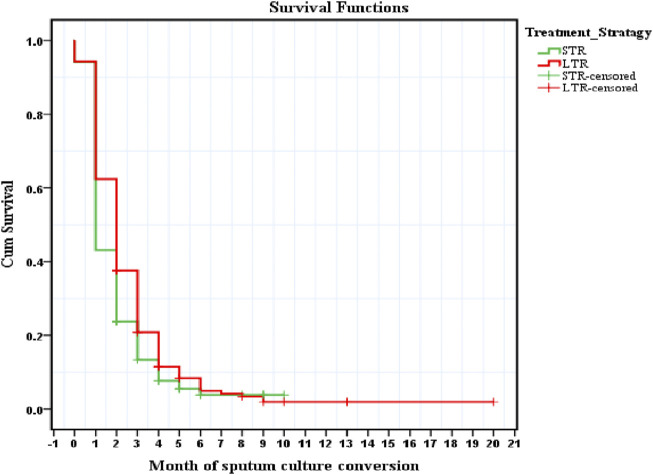
Time to sputum culture conversion among MDR-TB patients receiving Longer and Shorter treatment regimen.

In order evaluate time to SCC and predictors of early conversion, a total of 641 patients who were sputum culture positive at the baseline visit, had received treatment for at-least 2 months and were not LTFU prior to SCC were included in the final analysis. Among them, 272 (93.8%) patients in STR and 331 (94.3%) in LTR achieved SCC during their course of treatment (*p*-value = 0.786). Among those who achieved SCC in STR group, a total 81.9% were SC second month of treatment, while this proportion was 66.04% among those treated with LTR. Time to achieve SCC was significantly shorter in STR group than in LTR group. The results of Kaplan-Meier analysis revealed that the mean time to SCC was significantly shorter, (mean: 2.03 months, 95% CI: 1.79–2.26) in patients treated with STR, compared with those who were treated with LTR (mean: 2.69 months, 95% CI: 2.35–3.03) (*p*-value < 0.001, Log-rank test) (Figure 2).

In multivariate Cox proportional hazards regression analysis, the type of treatment regimen received emerged as the only predictor of time to SCC. Treatment with LTR had a statistically significant negative association with early SCC [adjusted Hazards ratio (HR) = 0.815 (0.692–0.959), *p*-value = 0.014] (Table 2).

**TABLE 2:** Predictors of time to sputum culture conversion in MDR-TB patients N = 641.

Variable	SCC		HR (95%CI)	*p*-value	aHR (95%CI)	*p*-value
	No	Yes	Univariate analysis		Multivariate analysis	
	No (%)	No (%)				
Gender						
Female	13 (4.0)	311 (96.0)				
Male	25 (7.9)	292 (92.1)	0.916 (0.781–1.075)	0.283		
Age						
<20	7 (4.5)	147 (95.5)				
21–40	16 (5.4)	281 (94.6)	0.950 (0.778–1.160)	0.614		
41–60	8 (5.6)	136 (94.4)	1.018 (0.806–1.286)	0.879		
>60	7 (15.2)	39 (84.8)	0.861 (0.605–1.227)	0.408		
Smoking						
No	30 (5.3)	536 (94.7)				
Yes	8 (10.7)	67 (89.3)	0.838 (0.650–1.080)	0.172	0.842 (0.652–1.087)	0.187
Weight						
≤30 kg	2 (5.7)	33 (94.3)				
31–60 kg	32 (6.0)	503 (94.0)	1.011 (0.710–1.438)	0.953		
>60 kg	4 (5.6)	67 (94.4)	1.091 (0.719–1.657)	0.682		
Sputum smear						
Negative	8 (12.9)	54 (87.1)				
Scanty + 1	20 (5.4)	350 (94.6)	1.213 (0.910–1.617)	0.187	1.272 (0.953–1.700)	0.103
+2, +3	10 (4.8)	199 (95.2)	1.177 (0.871–1.591)	1.591	1.269 (0.933–1.725)	0.129
Comorbidity						
No	28 (5.4)	488 (94.6)				
Yes	10 (8.0)	115 (92.0)	0.974 (0.794–1.193)	0.797		
Lung’s cavity						
No lesion	5 (12.5)	35 (87.5)				
One zone	12 (4.9)	233 (95.1)	1.163 (0.815–1.659)	0.406		
2–3 zone	20 (5.8)	325 (94.2)	1.246 (0.879–1.767)	0.216		
Not available	1 (9.1)	10 (90.9)	1.208 (0.598–2.441)	0.598		
Previous TB history						
New	14 (7.5)	172 (92.5)				
Cat-I	19 (5.2)	347 (94.8)	1.103 (0.918–1.324)	0.295		
Cat-II	3 (6.4)	44 (93.6)	0.903 (0.648–1.258)	0.546		
Unknown	2 (4.8)	40 (95.2)	0.956 (0.677–1.350)	0.798		
E Resistance						
No	34 (6.3)	505 (93.7)				
Yes	4 (3.9)	98 (96.1)	0.808 (0.650–1.004)	0.054	0.831 (0.654–1.055)	0.128
Z Resistance						
No	30 (6.1)	464 (93.9)				
Yes	8 (5.4)	139 (94.6)	0.871 (0.721–1.054)	0.155	0.916 (0.744–1.127)	0.406
S Resistance						
No	36 (6.3)	536 (93.7)				
Yes	2 (2.9)	67 (97.1)	0.879 (0.681–1.133)	0.319		
Resist to 5 FLD						
No	38 (6.1)	581 (93.9)				
Yes	0 (0.0)	22 (100.0)	0.879 (0.681–1.133)	0.319		
Treatment regimen						
STR	18 (6.2)	272 (93.8)				
LTR	20 (5.7)	331 (94.3)	0.808 (0.687–0.949)	**0.010**	0.815 (0.692–0.959)	**0.014**

TB, tuberculosis; STR, shorter treatment regimen; LTR, longer treatment regimen; FLD, first line drugs; Z, pyrazinamide; E, ethambutol; S, streptomycin.

### Treatment outcomes and predictors of unsuccessful treatment outcomes

The treatment outcomes of current study participants are given in Table 3. The proportions of patients who achieved successful treatment outcomes in STR and LTR groups were respectively (*n* = 262/313, 83.7%) and (*n* = 284/388, 73.2%) (*p*-value < 0.001). The proportion of cured patients was significantly higher in STR group (*n* = 250/313, 79.9%) than in LTR group (*n* = 275/388, 70.9%) (*p*-value = 0.006), whereas the proportion of deaths was significantly higher in LTR group (*n* = 71/388, 18.3%) than in STR group (*n* = 31/313, 9.9%) (*p*-value = 0.002). Among the 71 patients who died in the LTR group, 59 (83%) died in the initial 12 months of treatment. No significant difference between the two groups were observed in proportions of patients who were declared treatment completed, failed and LTFU (*p*-value > 0.05).

**TABLE 3:** Treatment outcomes of STR and LTR.

Treatment outcomes	STR N = 313	LTR N = 388	Total N = 701	*p*-value
	No (%)	No (%)	No (%)	
Successful	**262 (83.7)**	**284 (73.2)**	**546 (77.9)**	**0.001**
Completed	12 (3.8)	9 (2.3)	21 (3.0)	0.242
Cured	250 (79.9)	275 (70.9)	525 (74.9)	**0.006**
Unsuccessful	**51 (16.3)**	**104 (26.8)**	**155 (22.1)**	**0.001**
Failed	4 (1.3)	6 (1.5)	10 (1.4)	0.766
Died	31 (9.9)	71 (18.3)	102 (14.6)	**0.002**
Last to follow up	16 (5.1)	27 (7.0)	43 (6.1)	0.311

STR, shorter treatment regimen; LTR, longer treatment regimen.

In multivariate binary logistic regression analysis, patient’s age of 41–60 (OR = 2.35, *p*-value 0.004), > 60 years (OR = 4.70, *p*-value <0.001), baseline body weight of 31–60 (OR = 0.35, *p*-value = 0.005) and 60 kg (OR = 0.25, *p*-value <0.004) and treatment with LTR (OR = 1.91, *p*-value = 0.001) had statistically significant association with unsuccessful treatment outcomes (Table 4). This model fit was based on non-significant Hosmer-Lemeshow test (*p*-value = 0.379) and overall classification percentage (78.2%) from classification table (Table 4).

**TABLE 4:** Risk factors associated with unsuccessful treatment outcomes in MDR-TB patients.

Variable	Unsuccessful outcomes		OR (95%CI)	*p*-value	OR (95%CI)	*p*-value
	No	Yes	Univariate analysis		Multivariate analysis	
	No (%)	No (%)				
Gender						
Female	283 (79.5)	73 (20.5)				
Male	263 (76.2)	82 (23.8)	1.209 (0.846–1.728)	0.298		
Age						
<20	139 (83.2)	28 (16.8)				
21–40	263 (82.4)	56 (17.6)	1.057 (0.642–1.739)	0.827	1.227 (0.724–2.080)	0.448
41–60	114 (70.4)	48 (31.0)	2.090 (1.233–3.543)	0.006	2.351 (1.312–4.215)	**0.004**
>60	30 (56.6)	23 (43.4)	3.806 (1.932–7.499)	0.000	4.704 (2.270–9.752)	**0.000**
Smoking						
No	485 (78.2)	135 (21.8)				
Yes	61 (75.3)	20 (24.7)	1.178 (0.687–2.021)	0.552		
Weight						
≤30 kg	28 (63.6)	16 (36.4)				
31–60 kg	455 (78.6)	124 (21.4)	0.477 (0.250–0.909)	0.025	0.369 (0.184–0.739)	**0.005**
>60 kg	63 (80.8)	15 (19.2)	0.417 (0.181–0.959)	0.039	0.257 (0.101–0.650)	**0.004**
Sputum smear						
Negative	54 (79.4)	14 (20.6)				
Scanty + 1	316 (80.2)	78 (19.8)	0.952 (0.503–1.802)	0.880		
+2, +3	176 (73.6)	63 (26.4)	1.381 (0.718–2.657)	0.334		
Comorbidity						
No	447 (79.4)	116 (20.6)				
Yes	99 (71.7)	39 (28.3)	1.518 (0.994–2.318)	0.053	1.240 (0.775–1.982)	0.369
Lung’s cavity						
No lesion	31 (75.6)	10 (24.4)				
One zone	209 (77.1)	62 (22.9)	0.920 (0.427–1.980)	0.830		
2–3 zone	297 (79.2)	78 (20.8)	0.814 (0.383–1.732)	0.594		
Not available	9 (64.3)	5 (35.7)	1.722 (0.467–6.351)	0.414		
Previous TB history						
New	153 (72.9)	57 (27.1)				
Cat-I	314 (79.9)	79 (20.1)	0.675 (0.456–0.999)	0.049	0.673 (0.446–1.016)	0.060
Cat-II	44 (84.6)	8 (15.4)	0.488 (0.217–1.100)	0.084	0.614 (0.261–1.445)	0.264
Unknown	35 (76.1)	11 (23.9)	0.844 (0.401–1.773)	0.654	0.850 (0.930–1.850)	0.682
E Resistance						
No	462 (78.7)	125 (21.3)				
Yes	84 (73.7)	30 (26.3)	1.320 (0.832–2.094)	0.238		
Z Resistance						
No	422 (78.4)	116 (21.6)				
Yes	124 (76.1)	39 (23.9)	1.144 (0.756–1.732)	0.524		
S Resistance						
No	482 (77.0)	144 (23.0)				
Yes	64 (85.3)	11 (14.7)	0.575 (0.295–1.120)	0.104	0.484 (0.384–1.872)	0.684
Resist to all 5 FLD						
No	524 (77.4)	153 (22.6)				
Yes	22 (91.7)	2 (8.3)	0.311 (0.072–1.339	0.117	0.410 (0.077–2.195)	0.298
Treatment regimen						
STR	262 (83.7)	51 (16.3)				
LTR	284 (73.2)	104 (26.8)	1.881 (1.293–2.737)	**0.001**	1.918 (1.299–2.833)	**0.001**

TB, tuberculosis; STR, shorter treatment regimen; LTR, longer treatment regimen; FLD, first line drugs; Z, pyrazinamide; E, ethambutol; S, streptomycin.

## Discussion

To the best of our knowledge, this is the first study from Pakistan an MDR-TB high burden country, which evaluated the comparative effectiveness of LTR and STR among those MDR-TB patients who had no documented evidence of any SLD resistance and had no previous treatment with any SLD for ≥ 1 month. Our results demonstrated that STR was superior to LTR in terms of achieving early SCC. This finding was in line with the previous studies conducted elsewhere. For example, a study from Kyrgyzstan has reported that there was higher 1-month SCC among patients treated with STR than those who were treated with LTR (Zhdanova et al., 2021). Likewise, an almost two-fold greater likelihood of SCC by 2 months following treatment of MDR-TB patients with STR has been reported from Uzbekistan (du Cros et al., 2017). Moreover, in the current study, the emergence of STR as the only predictor of early SCC further strengthened the current finding of positive association between STR and early SCC.

We found that a total of 83.7% patients in STR group achieved successful treatment outcomes. This was in compliance with the pooled treatment success rate (80%) among 2,625 MDR-TB patients treated with STR (Abidi et al., 2020), observed in an IPD meta-analysis (83%) (Khan et al., 2017), nine African countries (82%) (Trébucq et al., 2018) and Bangladesh (85%) (Van Deun et al., 2010) but lower than that reported from Niger (89.2%) (Piubello et al., 2014) and Cameroon (89.3%) (Kuaban et al., 2015). The current treatment success rate in LTR group (73.2%) was in line with the pooled treatment success rate of 75.3% among LTR treated MDR-TB patients (*n* = 13,014) who were neither resistant to SLD nor had a history of treatment with any SLD ≥ 1 month (Abidi et al., 2020). In the present study, treatment with STR emerged as a predictor of successful treatment outcome. The remarkable difference in the treatment success rates between the two groups was due to significantly high proportion of cured patients in the STR group (79.9%) than in the LTR group (70.9%). Early SCC and high cure rate with STR reflected its superior anti-microbial activity against MDR-TB. As fluoroquinolones (FQs) are considered the backbone of MDR-TB treatment, the presence *Mfx* in STR which is comparatively more potent and effective than *Lfx* (present in LTR) against both susceptible and resistant MTB strains (Maitre et al., 2017; Du et al., 2019) could be one of the potential causes of early SCC and better cure rate in patients treated with STR. The presence of strong bactericidal agents like CFZ, and high dose INH which can retain activity against INH resistance due to inhA or katG 315 mutations could be the other possible cause of superior anti-microbial activity by STR (Van Deun et al., 2018; Du et al., 2019; Zhdanova et al., 2021). In current study, the mortality rates in the STR (9.9%) and the LTR (18.3%) were in compliance with the elsewhere reported mortality ranges for MDR-TB patients treated with STR (5.6%–7.8%) (Aung et al., 2014; Kuaban et al., 2015; Khan et al., 2017; Trébucq et al., 2018) and LTR (9.5%–25%) (Ahmad et al., 2015; Rao et al., 2015; Javaid et al., 2017c; Khan et al., 2019; Khan, et al., 2022a). However, in current study the mortality rate was significantly higher in the LTR group than the STR group. As in the current study, 15.2% (*n* = 59/388) patients in the LTR group died in the initial 12 months of treatment (vs. 9.9% mortality in the STR group), this advocates that the potential greater likelihood of death with lengthier duration of treatment was not the cause of higher mortality in patients treated with the LTR group (Abidi et al., 2020). We found no significant difference in the proportions of LTFU patients treated with STR (5.1%) and LTR (7.0%). This was in contradiction with the finding of an IPD meta-analysis which has evaluated the comparative effectiveness of STR and LTR among MDR-TB patients who were neither resistant to SLD nor had a previous history of treatment with any SLD *≥* 1 month (Abidi et al., 2020). The said study found a significantly high rate of LTFU among patients treated with LTR (14.6%) than those treated with STR (4.2%) (Abidi et al., 2020). However, in compliance with our finding the STREAM trial did not find any significant difference in proportion of LTFU patients treated with STR and LTR (Nunn et al., 2019). All patients of the current study were treated free of cost under similar programmatic conditions and were provided monthly food rations, transportation allowance to both patients and their treatment supporters, and patients’ psychological counselling on monthly visits. This signifies that receiving treatment under similar programmatic conditions with better patient support and backing during treatment could reduce LTFU rate in MDR-TB patients irrespective of regimen composition (Nunn et al., 2019). In multivariate analysis the emergence of patients age of 41–60 and > 60 years as predictors of unsuccessful end treatment outcomes among MDR-TB patients is in line with the previous studies (Kurbatova et al., 2012; Ahmad et al., 2015; Khan et al., 2019; Atif et al., 2020; Wahid et al., 2021). The concomitant presence of multiple factors like general physical deterioration, compromised immunity, multiple comorbidities, and complex medication schedule make older patients more likely to develop unsuccessful treatment outcomes (Kurbatova et al., 2012; Ahmad et al., 2015; Khan et al., 2019; Atif et al., 2020; Wahid et al., 2021; Khan et al., 2022b). In the current study, those patients who had a baseline body weight of > 30 kg were significantly less likely to develop unsuccessful treatment outcomes than their counterparts with a baseline body weight of <30 kg. Malnutrition or patients’ low body weight at baseline visit has been widely reported as a risk factor for unsuccessful treatment outcomes among MDR-TB patients. This association could be due to the sub-therapeutic concentrations of oral anti-TB drugs caused by poor gastrointestinal absorption and possible under-dosing in underweight patients (Ahmad et al., 2015; Khan et al., 2019; Wahid et al., 2021).

This study included MDR-TB patients diagnosed and treated in line with the guidelines recommended protocols at multiple PMDT units and their data were collected from the standardized ENRS. There was no statistically significant difference in patients of both groups in terms of gender, age, smoking, baseline weight and sputum smear grading, comorbidity, lungs lesions, previous TB treatment category and resistance to FLD. However, being a historical control study, some of the confounders may have remained obscure below the radar. Therefore, its results should be interpreted with some notable limitations like retrospective observational design, lack of information about the occurrence of adverse events and their impact on treatment outcomes and the absence of post-treatment follow-up to ensure the absence of relapses among the patients. Furthermore, patients who have been treated later (STR group) might have benefited from the experience of the healthcare personnel and program.

## Conclusion

Our results demonstrate that when compared with LTR, treatment with STR resulted in significantly earlier SCC, high cure and lower death rates among those MDR-TB patients who had no documented resistance and history of treatment with any SLD ≥ 1 month and were matched for gender, age, body weight, smoking status, disease severity, microbial load, comorbidity, drug resistance pattern and previous TB treatment regimen. After adjusting for confounders, treatment with LTR, patients’ age of > 40 years and baseline body weight of <30 kg emerged as predictors of unsuccessful treatment outcomes. It is important to mention that after the recent evidence and recommendations by WHO guidelines (Ahmad et al., 2018; WHO, 2019) the treatment of eligible MDR-TB patients with oral shorter regimen in which SLI in the STR is replaced by bedaquiline has been adopted by NTP in Pakistan. Therefore, it is suggested to evaluate the comparative effectiveness and safety of both shorter regimens.

## Data Availability

The original contributions presented in the study are included in the article/Supplementary Material, further inquiries can be directed to the corresponding author.
