# Supplementary material for: Comparative effectiveness of individualized longer and standardized shorter regimens in the treatment of multidrug resistant tuberculosis in a high burden country
Source: Front Pharmacol. 2022 Sep 6;13:973713. doi: 10.3389/fphar.2022.973713 (PMC9503836; doi:10.3389/fphar.2022.973713)
Supplement: Supplementary file 1 [file DataSheet1.PDF]

**Table S1: PMDT site wise distribution of study participants**

| S. No | PMDT site | Total No (%) | STR No (%) | LTR No (%) |
|---|---|---|---|---|
| 1 | District Head Quarter Hospital Faisalabad | 149 | 77 (51.7) | 72 (48.3) |
| 2 | Jinnah Hospital Lahore | 57 | 25 (43.9) | 32 (56.1) |
| 3 | Lady Reading Hospital Peshawar KPK | 144 | 60 (41.7) | 84 (58.3) |
| 4 | Nishter Hospital Multan | 141 | 51 (36.2) | 90 (63.8) |
| 5 | Medical Teaching Institute Mardan | 17 | 17 (100.0) | 0 (0.0) |
| 6 | Saidu Teaching Hospital Swat | 26 | 12 (46.2) | 14 (53.8) |
| 7 | Rawalpindi Leprosy Hospital Rawalpindi | 94 | 32 (34.0) | 62 (66.0) |
| 8 | Sheikh Zaid Hospital Rahimyar Khan | 73 | 39 (53.4) | 34 (46.6) |
| | **Total** | **701 (100.0)** | **313 (44.7)** | **388 (55.3)** |

PMDT; Programmatic management of drug resistant tuberculosis, KPK; Khyber Pukhtoonkhwa
STR; Shorter treatment regimen, LTR; Longer treatment regimen
